# Evaluation of a portfolio-based course on self-development for pre-medical students in Korea

**DOI:** 10.3352/jeehp.2019.16.38

**Published:** 2019-12-11

**Authors:** Dong Mi Yoo, A Ra Cho, Sun Kim

**Affiliations:** Department of Medical Education, College of Medicine, The Catholic University of Korea, Seoul, Korea; Hallym University, Korea

**Keywords:** Medical education, Portfolio, Self-development, Social competence, Korea

## Abstract

**Purpose:**

We have developed and operated a portfolio-based course aimed at strengthening pre-medical students’ capabilities for self-management and self-improvement. In order to determine the effectiveness of the course and to establish future operational strategies, we evaluated the course and the students’ learning experience.

**Methods:**

The subjects of this study were 97 students of a pre-medical course “Self-development and portfolio I” in 2019. Their learning experience was evaluated through the professor’s assessment of portfolios they had submitted, and the program was evaluated based on the responses of 68 students who completed a survey. The survey questionnaire included 32 items. Descriptive statistics were reported for quantitative data, including the mean and standard deviation. Opinions collected from the open-ended question were grouped into categories.

**Results:**

The evaluation of students’ portfolios showed that only 6.2% of the students’ portfolios were well-organized, with specific goals, strategies, processes, and self-reflections, while most lacked the basic components of a portfolio (46.4%) or contained insufficient content (47.4%). Students’ responses to the survey showed that regular portfolio personality assessments (72.1%), team (64.7%), and individual (60.3%) activities were felt to be more appropriate as educational methods for this course, rather than lectures. Turning to the portfolio creation experience, the forms and components of the portfolios (68.2%) and the materials provided (62.2%) were felt to be appropriate. However, students felt that individual autonomy needed to be reflected more (66.7%) and that this course interfered with other studies (42.5%).

**Conclusion:**

The findings of this study suggest that standardized samples, guidelines, and sufficient time for autonomous portfolio creation should be provided. In addition, education on portfolio utilization should be conducted in small groups in the future.

## Introduction

### Background

Physicians should be equipped with social competence, which includes professionalism, understanding of oneself and others, self-management, leadership, and communication, in addition to the clinical competence necessary to treat diseases. However, according to a survey conducted in Korea in 2013, the actual level of competence in practice was found to be very low compared to the recognition of the importance of social competence [1]. These results demonstrate that the medical education process has not yet succeeded in training physicians to have the required level of social competence [2].

Most of the survey respondents in that study reported that they did not have learning experiences in medical school on competencies such as objective self-assessment, time management, and self-improvement. Social competence, however, cannot be developed in a short period of time. Therefore, it is very important for students to develop social competence-related knowledge, skills, and attitudes at the start of their medical education. Then, they should have regular opportunities to apply their knowledge and skills, enabling them to make self-directed developments.

Accordingly, we developed a portfolio-based course aimed at building self-management and self-improvement capabilities among pre-medical students. Portfolios are records of evidence collected by students with clear learning goals; they are also records of learners’ sustained and systematic reflection on their progress and performance that can be utilized in various ways, ranging from basic education to professional development education [3,4].

As students create their portfolios, they play key roles in their own learning process; through evaluation, they acquire reflective thinking skills, experience deep learning, and learn what they need for future learning. Portfolio creation is a specific tool used in health care education that allows students to discover their stand, what they lack, and what they need to do to be able to study by themselves. Portfolio creation has also been recognized for its educational value in terms of knowledge, skills, and attitudes [5,6].

### Purpose

We developed and operated a portfolio-based course for pre-medical students, aiming at strengthening their self-management and self-improvement capabilities. In order to examine the effects of this course, we conducted this study with the following goals. The first objective of the study was to investigate students’ learning experience through the professor’s evaluation of students’ portfolios. The second objective was to evaluate the adequacy of course operation, the portfolio creation experience, and students’ perceptions of the value of the course through a survey administered to students. Through this study, we aimed to enhance the value of education on portfolio utilization and to search for effective operational strategies for future applications.

## Methods

### Ethics statement

This study was approved by the Institutional Review Board of Songeui Medical Campus, the Catholic University of Korea (IRB approval no., MC19EESI0096). No informed consent forms were collected, but the participants were clearly informed of the purpose of this study, and were not pressured to participate in this study in any way. Therefore, there were no disadvantages of non-participation. A waiver of consent was also included in the IRB approval.

### Study design

This study was based on a survey.

### Subjects

The subjects of this study were 97 first-year pre-medical students (70 male students, 27 female students) who took the course “Self-development and portfolio 1.” The portfolios of the 97 students were evaluated by the professor in charge. Course evaluation data were obtained from questionnaires filled out by 68 students, excluding those who did not complete the survey.

### Setting

This course was a part of the regular curriculum at the Catholic University College of Medicine in the 2019 academic year. It was implemented as 9 classes (2 hours per class) from March to June of the first semester, as a required course (pass/non-pass) for all students.

**Fig. 1.**
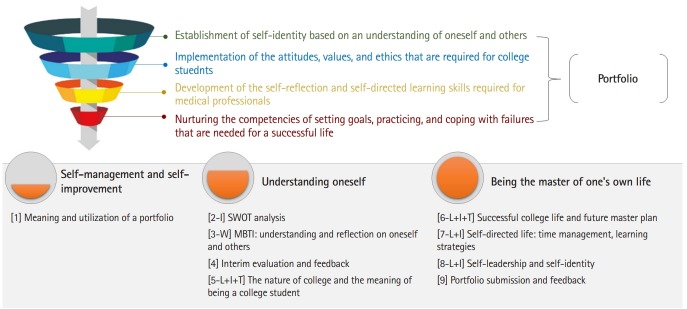
Educational goals of the portfolio-based course and outlines of the lessons. SWOT analysis, strengths, weaknesses, opportunities, and threats analysis; MBTI, Myers–Briggs Type Indicator; L, large lecture; W, workshop; I, individual activity; T, team activity.

The course was tailored to the learning goals of the pre-medical curriculum, and consisted of the following three domains: self-management and self-improvement, understanding of oneself, and being the master of one’s own life. Depending on the topics, the course material was delivered in various ways, including lectures, psychological tests, team or individual activities, and presentations. The portfolios were evaluated on a scale from A through D, and individual feedback was provided to the students in the form of a written evaluation by the professor in charge (Fig. 1). The evaluation form and criteria used are given in Supplement 1.

### Data collection

After completion of the portfolio-based course, the students’ learning experience and responses to the course evaluation questionnaire were analyzed in order to determine whether the course should be continued to be offered and to identify areas for development. The students’ learning experience was assessed by evaluating their portfolios, which constituted a summary of their overall learning, and the portfolios were evaluated immediately after each class. In order to exclude the possibility of inconsistency generated by different evaluators, only the professor in charge of the course evaluated the students, but the researcher and the professor agreed in advance on the evaluation items (goals, processes, self-reflection, and portfolio creation) and specific criteria (Supplement 1). The course was evaluated using items that were developed for the purpose of this study in consultation with 2 medical education specialists, in addition to the regular evaluation items for all courses at the university level. The questionnaire was administered after the last class (Supplement 2), and it was comprised of 32 items, including evaluations of course management, the adequacy of the educational content, self-assessment, and the portfolio creation experience. The Cronbach α value was 0.946, and the original source data are given in Dataset 1.

### Statistics

Descriptive statistics were reported for quantitative data, including the mean and standard deviation. The specific replies collected from the open-ended question were grouped into categories. All data were interpreted through the consensus of 2 researchers in order to avoid biased or subjective views and data omission. The other researcher reviewed the initial work as an auditor.

## Results

### Portfolio evaluation

Based on the portfolio assessment using standardized criteria for feedback, as given in Supplement 1, only 6.2% of the portfolios had specific goals, effective strategies and processes for achieving their goals, and self-reflections (corresponding to a grade of A); 47.4% of the portfolios included all the key components but lacked specific and sufficient content (corresponding to a grade of B); and 46.4% of the portfolios lacked even basic components (corresponding to a grade of C).

### Course evaluation by students

#### Educational methods and contents

**Fig. 2.**
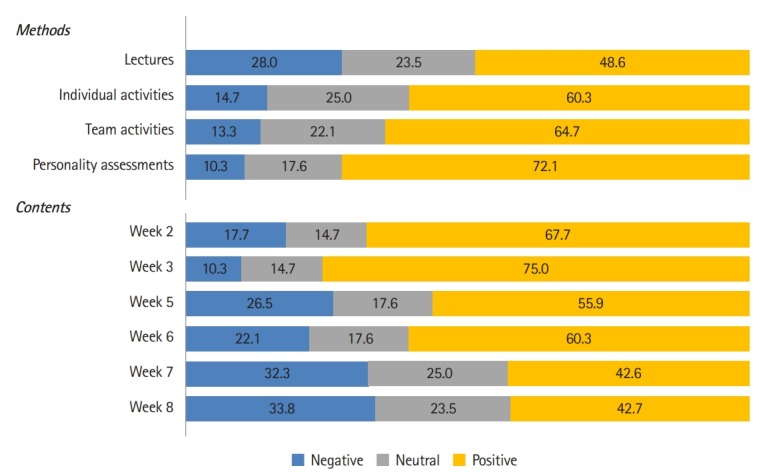
Evaluation of the course (%). Week 2: SWOT analysis activity; week 3: MBTI workshop; week 5: the nature of college and the meaning of being a college student; week 6: successful college life and future master plan; week 7: self-directed college life; week 8: self-leadership and self-identity. SWOT analysis, strengths, weaknesses, opportunities, and threats analysis; MBTI, Myers–Briggs Type Indicator.

Students indicated that examinations (72.1%), team activities (64.7%), and individual activities (60.3%) were more appropriate methodologies for the portfolio-based course than lectures alone (Fig. 2). High levels of satisfaction were reported for the second-week class (strengths, weaknesses, opportunities, and threats [SWOT], 67.7%), in which the students attempted to explore their own strengths, weaknesses, opportunities, and threats through individual analysis activities, and for the third-week class (Myers–Briggs Type Indicator, 75.0%), in which understanding and reflection on oneself and others was promoted through teamwork (SWOT).

In contrast, low levels of satisfaction were reported for the seventh-week class (42.7%), which dealt with self-directed life, and the eighth-week class (42.6%), which focused on self-leadership, self-identity. One of the responses to the open-ended question read, “Since students who are competent to enter medical school must already have their own strategies for self-directed learning, time management, learning methods, and self-leadership, lectures related to such topics are not necessary.”

#### Portfolio creation experience and achievements

**Fig. 3.**
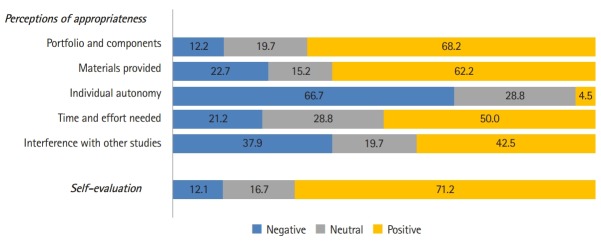
Evaluation of the portfolio creation experience (%).

The forms and components of the portfolios, the personality test profiles, and the worksheets that were provided during class were evaluated as appropriate (Fig. 3). However, different opinions were expressed regarding the level of time and effort needed by each individual, as 66.7% of the respondents replied that individual autonomy should be reflected more, and 42.5% of students indicated that the portfolio-based course interfered with other studies. Some exemplary replies to the open-ended questions were: “The class was too long to be efficient. Insufficient time was allocated for creating the portfolio.” “After we set our goals and plans, there was not enough time to implement them due to the tight submission deadline.”

**Fig. 4.**
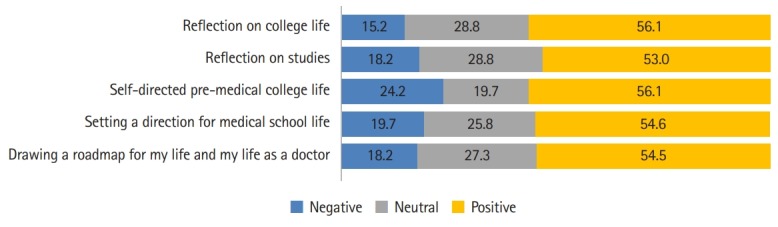
Evaluation of the achievements (%).

In addition, the students replied that portfolio creation was helpful in terms of reflection on studies (53.0%), reflection on their college life (56.1%), self-directed pre-medical college life (56.1%), setting the direction of their medical school life (54.6%), and in drawing a roadmap for their lives and their lives as doctors (54.5%); these replies accounted for 54.9% of all respondents on average (Fig. 4). For open-ended questions, some exemplary positive replies were as follows: “I was given the opportunity to look back and design my life through tasks.” “I was able to look back at my past and make future self-development plans.” However, on average, 19.1% of respondents stated that portfolio creation was not helpful for them. Since the average score was 3 points and within 1 standard deviation, perceptions of the value of the course varied among individuals.

#### Educational method using portfolios

Only 15.2% of students indicated that a large group of 60 or more would be their preferred portfolio coaching method; instead, most students responded that groups of 10 or fewer (53.0%) and one-on-one setting (31.8%) would be more appropriate. An illustrative reply stated, “It would be better to present a detailed guideline for the portfolio at the beginning of the semester, and then let each student create a portfolio first without taking the class. The portfolio would be submitted at the end of the semester, and then one-on-one feedback would be provided.”

## Discussion

### Key results

We developed and operated a portfolio-based course to improve pre-medical students’ self-management and self-improvement ability. Although 71.2% of students replied that they worked hard and took creation of their portfolios seriously, only 6.2% among them earned a grade of A. However, 54.9% of the students responded that their portfolio creation experience helped them to reflect on and plan the rest of their university life and even their subsequent life as a doctor, confirming its educational value.

### Interpretation and suggestion

In order to achieve better outcomes, the following aspects were identified as needing improvement in terms of portfolio management methods, curriculum and educational methods, and the process of portfolio creation:

First, only a small percentage of students received a grade of A. Most students lacked an understanding of the concepts underlying a portfolio, the meaning of a portfolio, and how to utilize them, and their portfolios often lacked basic components. Therefore, although providing autonomy for portfolio creation is necessary, it might also be effective to provide standard samples, specific guidelines, and various worksheets. In addition, developing pre-training and giving specific feedback on utilizing portfolios will help students gain an accurate understanding of the concepts underlying portfolios and the meaning of a portfolio.

Second, it is necessary to improve the curriculum and educational methods. Pre-medical students are adult learners. As many students indicated, they are likely to already have established personal approaches to concepts related to self-directed college life (e.g., learning strategies and time management), self-leadership, and self-identity. Therefore, it may be redundant for the instructor to spend time lecturing about this content, and it would be better to allot more time for portfolio creation instead. Negative evaluations by students tended to increase as the semester progressed, which implies that portfolio creation may require a considerable amount of time and that the workload may cause fatigue. Correspondingly, 42.5% of the students replied that portfolio creation interfered with their other studies, and similar opinions were given in replies to the open-ended question. This problem can be solved by reducing the amount of lecture time and allotting time for autonomous portfolio creation within the regular classes.

Third, portfolio-based courses should be implemented in small-group or one-on-one settings, as 84.8% of the students responded that it would be appropriate for the course to be administered to small groups. Considering that this course focuses on individual reflection and self-improvement, making it necessary to implement a form of process-oriented education, it is inevitably difficult to maximize the value of education in large groups. However, if small groups or a one-to-one setting is adopted, the number of instructors needed will increase, because it will be necessary to deliver the course to different groups at the same time. Therefore, any university program interested in trying portfolio-based education should secure sufficient manpower through instructor training. Additionally, it is necessary to develop and standardize portfolio forms, the writing process, evaluation criteria, and feedback items to ensure the quality of the entire educational process and how it is evaluated.

### Comparison with previous studies

It is only recently that portfolios have been introduced into undergraduate education, and there are not enough previous studies on the introduction of the portfolio concept into pre-medical courses in other countries. In addition, the use of different measurement tools makes it difficult to make direct comparisons. Nevertheless, previous studies have reported that portfolios are seen as increasingly valuable in medical education, and that the systemic use of portfolios increases students’ reflections on their own experiences and professional competence. In addition to students’ learning, we anticipate that portfolios will support their educational achievements. For example, in Saudi Arabia, the portfolio method was implemented for medical students, and self-assessment of the skills learned through this process was conducted. Evidence was found that this process was valued positively and that portfolio-based learning may be becoming more accepted by students [7]. Web-based electronic portfolio creation was implemented for Spanish medical students to evaluate their knowledge of manual skills and surgical techniques. The students stated that portfolio creation was useful when the workload and complexity of learning objectives were adequate, and that the electronic portfolio helped them to set directions for their learning [8]. In the United Kingdom, electronic portfolio creation for medical students was implemented and a qualitative analysis was performed on the usefulness of this process based on students’ opinions obtained through free writing. Students showed interest in 5 aspects: the objectives of portfolio creation, usage, acceptance, strengths and limitations of portfolio utilization, and the impact of portfolios on professional identity and learning. Portfolios also had a positive influence on understanding one’s identity [9]. Lund University, in Sweden, has implemented a portfolio of standardized general practice guidelines for senior medical students. The standardized portfolio provided ample opportunities for experts to evaluate students’ professionalism, and support from mentors and examiner interviews contributed to students’ proper implementation of the portfolio [10].

### Limitation

It is difficult to generalize the results of this study, which is based only on the experiences of a single medical school. In addition, since the course was offered for only 1 semester, the effectiveness of the curriculum could not be verified objectively.

### Conclusion

Despite the limitations of this study, students’ perceptions and experiences provided important insights into the value of this portfolio-based course. Therefore, this study is meaningful in that the value and possibility of a portfolio-based course was confirmed through students’ evaluations. In addition, basic principles for developing a specific curriculum and educational methods were proposed. If the course is revamped based on this study, if mid- and long-term operational experience is gathered in subsequent years, and if students become habituated to portfolio creation, future research would yield more meaningful insights into its educational effects.

## Footnotes

**Data availability** Data files are available from Harvard Dataverse: https://doi.org/10.7910/DVN/I8BWYM Dataset 1. Raw data of the survey used for course evaluation.

## Supplementary materials

Data files are available from Harvard Dataverse: https://doi.org/10.7910/DVN/I8BWYM

Supplement 1. Portfolio evaluation criteria and standard feedback contents.

Supplement 2. Course evaluation survey tool.

Supplement 3. Audio recording of the abstract.

## References

[1] Kim CJ, Kwon I, Han HJ, Heo YJ, Ahn D (2014). Korean doctors’ perception on doctor's social competency: based on a survey on doctors. J Korean Med Assoc.

[2] Dent JA, Harden RM (2013). A practical guide for medical teachers.

[3] Friedman Ben David M, Davis MH, Harden RM, Howie PW, Ker J, Pippard MJ (2001). AMEE Medical Education Guide no. 24: portfolios as a method of student assessment. Med Teach.

[4] Kim KJ (2014). e-Portfolios for learning and assessment in medical education. Korean Med Educ Rev.

[5] Vance GH, Burford B, Shapiro E, Price R (2017). Longitudinal evaluation of a pilot e-portfolio-based supervision programme for final year medical students: views of students, supervisors and new graduates. BMC Med Educ.

[6] Buckley S, Coleman J, Davison I, Khan KS, Zamora J, Malick S, Morley D, Pollard D, Ashcroft T, Popovic C, Sayers J (2009). The educational effects of portfolios on undergraduate student learning: a Best Evidence Medical Education (BEME) systematic review: BEME Guide no. 11. Med Teach.

[7] Fida NM, Hassanien M, Shamim MS, Alafari R, Zaini R, Mufti S, Al-Hayani A, Farouq M, Al-Zahrani H (2018). Students' perception of portfolio as a learning tool at King Abdulaziz University Medical School. Med Teach.

[8] Sanchez Gomez S, Ostos EM, Solano JM, Salado TF (2013). An electronic portfolio for quantitative assessment of surgical skills in undergraduate medical education. BMC Med Educ.

[9] Belcher R, Jones A, Smith LJ, Vincent T, Naidu SB, Montgomery J, Haq I, Gill D (2014). Qualitative study of the impact of an authentic electronic portfolio in undergraduate medical education. BMC Med Educ.

[10] Haffling AC, Beckman A, Pahlmblad A, Edgren G (2010). Students’ reflections in a portfolio pilot: highlighting professional issues. Med Teach.

