# Supplementary material for: Evaluation of a portfolio-based course on self-development for pre-medical students in Korea
Source: J Educ Eval Health Prof. 2019 Dec 11;16:38. doi: 10.3352/jeehp.2019.16.38 (PMC7040426; doi:10.3352/jeehp.2019.16.38)
Supplement: Supplementary file 2 — Supplement 1. Portfolio evaluation criteria and standard feedback contents. [file jeehp-16-38-suppl1.pdf]

Supplement 1. 포트폴리오 평가 준거 및 스탠다드 피드백 컨텐츠

영역별 평가 준거	
목표설정	1. 제시한 학습성과를 포함하고 있는가
	2. 자신에게 가치가 있는가
	3. 실현가능하고 현실적인가
	4. 행동에 옮길 수 있도록 구체적인가
학습계획 및실행	5. 목표에 맞는 활동과 학습을 실천하였는가
	6. 활동과 학습의 내용이 구체적으로 충실하게 기술되었는가
자기성찰	7. 목표의 표면적 달성 여부에만 그치지 않고 내면까지 돌아보았는가
	8. 자신의 학습과정과 내용에 대한 강점과 약점을 살펴보았는가
	9. 반성에 그치는 것이 아니라 스스로 개선방법과 전략을 도출하였는가
포트폴리오 작성	10. 기본양식을 준수하고 포트폴리오로서 구성력을 갖추었는가
	11. 적절한 문장과 용어의 사용으로 표현력을 갖추었는가
	12. 관련 학습자료와 질의 수준이 적절한가
평가등급별 피드백	
A	☞ 무엇을 달성하고 학습하고자 하는지 목표가 구체적이고 뚜렷함
	☞ 목표를 달성하기 위한 전략과 학습과정, 탐구과정 등이 전반적으로 잘 진행되었고 포트폴리오에 잘 정리되었음
	☞ 포트폴리오에 자기성찰의 경험이 잘 드러남
B	☞ 포트폴리오의 구성요소를 빠진 부분 없이 잘 작성하였지만 각 구성요소의 작성 내용이 구체적이지 않고 부족함
	☞ 자기계발 포트폴리오의 형태는 갖추었으나 일부 구성요소에 미비한 부분이 존재함
C	☞ 기본적인 포트폴리오 구성요소를 갖추지 못함
	☞ 포트폴리오 개념, 활용 방법에 대한 이해가 부족함
	☞ 전반적으로 포트폴리오 작성 및 활용도를 높이기 위한 노력이 필요함
F	☞ 평가 불가
