# Supplementary material for: Evaluation of a portfolio-based course on self-development for pre-medical students in Korea
Source: J Educ Eval Health Prof. 2019 Dec 11;16:38. doi: 10.3352/jeehp.2019.16.38 (PMC7040426; doi:10.3352/jeehp.2019.16.38)
Supplement: Supplementary file 3 — Supplement 2. Course evaluation survey tool. [file jeehp-16-38-suppl2.pdf]

Supplement 2. 프로그램 평가 설문툴

번호	문항	응답
교과목 운영 전반: 운영 방식, 교육방법, 교육내용의 적절성 등에 대한 평가		
1	나는 수업 전에 이미 포트폴리오의 개념과 특성에 대해 알고 있었다.	①②③④⑤
2	오리엔테이션은 수업의 전반적인 과정을 이해하는데 도움이 되었다.	①②③④⑤
3	제시된 학습성과는 명확하였다.	①②③④⑤
4	시간별 과정은 짜임새 있고 순서 있게 구성되었고 생각한다.	①②③④⑤
5	매 과정의 시간과 분량은 적절하였다.	①②③④⑤
6	본 교과목의 내용과 활동, 체험은 내가 따라갈 수 있는 수준이었다.	①②③④⑤
7	교수는 학생들의 적극적인 참여와 관심을 유도하였다.	①②③④⑤
8	주어진 강의자료와 워크시트 등은 학습하는데 도움이 되었다.	①②③④⑤
(9~12) 아래의 방식은 포트폴리오 수업에 적합한 교육방법이었다.		
9	강의	①②③④⑤
10	개인 활동	①②③④⑤
11	팀 활동	①②③④⑤
12	다양한 검사	①②③④⑤
(13~18) 아래의 활동은 학습성과 달성에 도움이 되었다.		
13	SWOT : 나의 강점, 약점, 기회, 위협 탐색 등	①②③④⑤
14	MBTI: 나와 타인의 성격, 사고방식에 대한 이해와 관계 형성 등	①②③④⑤
15	대학의 본질과 대학생이 된다는 것의 의미: 대학생으로서의 성숙한 태도와 올바른 가치 형성 등	①②③④⑤
16	미래 마스터플랜 설계: 성공적인 대학생활을 위한 목표와 수행 전략 수립 등	①②③④⑤
17	자기주도적 대학생활:: 실패요인 분석을 통한 목표 설정, 학습전략, 시간관리 전략 수립 등	①②③④⑤
18	셀프리더십: 나의 학습동기 분석과 동기 강화 전략 수립 등	①②③④⑤
(19~20) 자기평가		
19	나는 주차별 과제 및 발표를 성실하게 준비하여 수행하였다.	①②③④⑤
20	나는 수업에 적극적으로 참여하였다.	①②③④⑤
포트폴리오 작성 경험과 성과: 스스로 포트폴리오를 구성하고 만들어 간 과제와 학습경험에 대한 평가		
21	대학생활 전반을 성찰하는데 도움이 되었다.	①②③④⑤
22	학업을 성찰하는데 도움이 되었다.	①②③④⑤
23	의예과 생활을 주도적으로 해 나가는데 도움이 되었다.	①②③④⑤
24	의과대학 생활에 대한 방향 설정에 도움이 되었다.	①②③④⑤
25	나의 삶, 의사로서의 삶에 대한 로드맵을 그리는데 도움이 되었다.	①②③④⑤
26	포트폴리오 양식은 필요한 요소로 잘 구성되었다.	①②③④⑤
27	제공된 수업자료는 포트폴리오 작성에 도움이 되었다(검사 결과 프로파일, 워크시트 등).	①②③④⑤
28	포트폴리오 내용, 작성 과정에 개인의 자율성이 더 반영되어야 한다고 생각한다.	①②③④⑤
29	포트폴리오 작성 과정에 필요한 시간과 노력은 적절한 수준이었다.	①②③④⑤
30	포트폴리오 작성으로 다른 학업에 방해를 받은 경험이 있다.	①②③④⑤
31	나는 성실하고 진지한 자세로 포트폴리오를 만들고 완성해 갔다.	①②③④⑤
32	다음 중 포트폴리오 피드백, 지도를 위해 선호하는 방식을 하나만 선택하여 V 표시하세요.	개인(일대일 멘토링)<br>그룹(10명 이하)<br>전체(60명 이상)

* Likert scale 1~5
